# Oxidative Stress and NLRP3 Inflammasome as Markers of Cardiac Injury Following Cardiopulmonary Bypass: Potential Implications for Patients with Preoperative Heart Failure with Reduced Ejection Fraction

**DOI:** 10.3390/antiox14111311

**Published:** 2025-10-30

**Authors:** Rodrigo L. Castillo, Rodrigo A. Carrasco, Alejandro Gonzaléz-Candia, Esteban G. Figueroa, Adolfo A. Paz, Alejandro A. Candia, Sawa Kostin, Nikolaos Pagonas, Pamela V. Arias, Emilio A. Herrera, Robert A. Pérez, Sebastián Iturra

**Affiliations:** 1 Departamento de Medicina Interna Oriente, Facultad de Medicina, Universidad de Chile, Santiago 7500922, Chile; 2 Unidad de Paciente Crítico, Hospital del Salvador, Santiago 8330068, Chile; 3 Department of Cardiology, University Heart Center, University Hospital Zurich, Univesity of Zurich, 8091 Zurich, Switzerland; rodrigo.carrascoloza@usz.ch; 4 Institute of Health Sciences, University of O’Higgins, Rancagua 3655000, Chile; alejandro.gonzalez@uoh.cl; 5 Escuela de Obstetricia, Facultad de Ciencias para el Cuidado de la Salud, Universidad San Sebastián, Santiago 7510157, Chile; esteban.figueroa@uss.cl; 6 Laboratory of Vascular Function and Reactivity, Pathophysiology Program, Institute of Biomedical Sciences (ICBM), Faculty of Medicine, Universidad de Chile, Santiago 8330015, Chile; adolfo.paz@ug.uchile.cl (A.A.P.);; 7 Department for the Woman and Newborn Health Promotion, Universidad de Chile, Santiago 8330015, Chile; alejandrocandiah@uchile.cl; 8 Faculty of Health Sciences, Brandenburg Medical School Theodor Fontane, 16816 Neuruppin, Germany; costin.sava@mhb-fontane.de; 9 Department of Cardiology, Medical School Theodor Fontane, University Hospital Ruppin-Brandenburg, 16816 Neuruppin, Germany; n.pagonas@ukrb.de; 10 International Center for Andean Studies (INCAS), Universidad de Chile, Putre 1970000, Chile; 11 School of Medicine, Universidad de los Andes, Santiago 7620001, Chile; raperez@miuandes.cl; 12 Instituto Nacional del Tórax, Santiago 7510150, Chile; sebastian.iturra@allina.com

**Keywords:** heart failure with reduced left ventricular ejection fraction, HF with preserved ejection fraction, NLRP3, 3-nitrotyrosine, oxidative stress markers, postoperative atrial fibrillation

## Abstract

Cardiopulmonary bypass (CPB) can lead to cardiac damage due to oxidative stress (OS) and inflammation in heart failure (HF). We tested the hypothesis that preoperative HF patients with reduced ejection fraction (HFrEF) subjected to CBP have higher levels of OS and NLRP3 (NOD-, LRR- and pyrin domain-containing protein 3) in heart and plasma and in those that develop postoperative AF (pAF) as a clinical outcome. HF was categorized for preoperative left ventricular EF: preserved (HFpEF > 50%, n = 27) and reduced EF (HFrEF ≤ 40%, n = 25). Samples of atrial tissue, pericardial fluid, and plasma were collected at surgery to assess NLRP3 expression; 3-nitrotyrosine (3-NT), thiobarbituric acid reaction (TBARS), and nuclear factor erythroid 2-related factor 2 (Nrf2) in atrial tissue; NLRP3, IL-1β, and IL-18 expression in pericardial fluid; and antioxidant capacity, 8-isoprostanes, and malondialdehyde (MDA) in plasma. Reactive oxygen species, 3-NT, and NLRP3 in atrial tissue were determined by immunohistochemistry in a subset of pAF patients. Plasma and atrial tissue 3-NT and MDA were higher in HFrEF compared with HFpEF. Lipid peroxidation products were higher in both plasma and atrial tissue in pAF (n = 29), compared to sinus rhythm (SR) (n = 23). In HFrEF patients, the values of tissue ROS, 3-NT, and NLRP3 were higher than in HFpEF patients. In addition, the expression levels of NLRP3, IL-1β, and IL-18 were higher in atrial tissue and pericardial fluid in HFrEF. Patients with preoperative HFrEF showed higher OS in plasma and the expression of NLRP3, ROS, and 3-NT in atrial tissue biopsies and pericardial fluid. This finding suggests a potential pharmacologic therapy for pAF and clinical complications due to CPB.

## 1. Introduction

During cardiac surgery with extracorporeal circulation, the use of cardiopulmonary bypass (CPB) can lead to myocardial injury due to multiple factors. Among these factors, oxidative stress (OS) and inflammation are significant contributors that may lead to contractile dysfunction by initiating a series of processes that may result in pathological remodeling. This cardiac remodeling involves a series of molecular, cellular, and interstitial changes that occur after injury, often manifesting clinically as alterations in size, mass, geometry, and heart function [1]. Interestingly, the molecular and cellular processes related to cardiac remodeling are more pronounced in patients with preoperative heart failure with reduced ejection fraction (HFrEF), which may increase the susceptibility of myocardial tissue to the effects induced by CPB. Understanding the potential bidirectional interactions between CPB and cardiac remodeling is crucial, as this could suggest a higher incidence of postoperative complications when both conditions occur simultaneously in patients.

Additionally, patients undergoing CBP may also have heart failure with preserved ejection fraction (HFpEF) as a previous condition. The functional capacity and quality of life in HFpEF patients are significantly impaired, leading to high morbidity and mortality rates [2], so this condition could represent an additional risk for patients who undergo CPB. However, to date, the interaction between CPB and cardiac remodeling in patients with HFpEF has not been thoroughly evaluated. Furthermore, patients undergoing CPB may also experience postoperative arrhythmias, including atrial fibrillation (AF), which can be self-limiting but also associated with increased morbidity, mortality, and healthcare costs. Postoperative atrial fibrillation (pAF) is a common complication of cardiac surgery with extracorporeal circulation, contributing to longer hospital stay, higher medical costs, and increased mortality rates [3]. Despite efforts to optimize anesthetic protocols, surgical techniques, and medical treatments, including the use of antiarrhythmic medications such as beta-blockers and amiodarone, the incidence of pAF remains notably high, ranging from 27% to 55% [4]. This highlights the need for new markers and pharmacological targets, given the suboptimal effectiveness of current perioperative pharmacological treatments. Although the exact pathophysiological cause of pAF remains unclear, it is believed to have multiple contributing factors. Potential causes of pAF include atrial dilatation, age-related fibrosis, cardiac structural damage, hypertension, and other comorbid conditions [5,6]. Certain patients, especially those with cardiovascular risk factors or specific types of cardiac surgery, such as valvular pathologies, may be more susceptible to developing AF postoperatively [7]. Additionally, from an electro-physiological perspective, the substrate could be a pre-existing condition associated with the development of heterogeneous refractoriness after surgery [8]. These events could be linked to imbalances in calcium homeostasis, which might activate reentry mechanisms [9].

In addition to the aforementioned risk factors and electrophysiological changes associated with pAF, significant inflammatory and oxidative stress mechanisms may also play a role at both molecular and cellular levels as a result of surgery and CPB [10]. Specifically, the surgical procedure may also contribute to the development of pAF due to operative trauma from dissection, tissue manipulation, and pericardial inflammation due to cell infiltration, such as that of peripheral blood mononuclear cells [9]. However, it remains unclear whether inflammation acts mainly as a systemic or local phenomenon reflecting an active inflammatory process within the heart [11,12]. The migration of various inflammatory cells can also trigger oxidative stress (OS) injury, increasing reactive oxygen species (ROS) production from their cellular sources [13]. Markers indicative of a prooxidant imbalance towards OS, such as malondialdehyde (MDA), carbonylated proteins, and lower levels of reduced glutathione (GSH), have been reported as a consequence of ROS burst during early reperfusion after cardiac surgery [14]. These cellular events related to OS and inflammatory processes could potentially be associated with pAF, rather than just being an epiphenomenon to cardiac surgery [15,16]. This has led some researchers to propose that ischemic time and the subsequent OS during early reperfusion could be potential triggers for pAF [16]. In this context, the oxidative burst may partially cause and determine electrical remodeling processes that trigger more significant reentry activity and, therefore, a greater susceptibility to developing AF and its perpetuation [17]. Our previous trial demonstrated a relationship between ischemia–reperfusion (IR) injury and oxidative modification in the atrial tissue of patients undergoing CPB [15]. Indeed, the incidence of pAF is associated with reperfusion time during surgery, which also influences the induction of certain ROS sources, such as NADPH oxidase [13]. In addition, an active cardiac remodeling process occurring in patients with HFrEF could indirectly affect oxidative balance and inflammation, leading to the occurrence of pAF in patients undergoing CPB [18].

However, the mechanisms linking the occurrence of pAF in patients with preserved LVEF who underwent CPB, and a potential substrate for cardiac remodeling, have not yet been well established. From a clinical perspective, some clinical trials have indicated a relationship between ventricular function and postoperative complications in patients undergoing coronary artery bypass grafting [19,20]. In terms of identifying potential predictors of pAF and cardiac remodeling, it has been clinically reported that subclinical atrial abnormalities, as assessed through preoperative left atrial strain measurements in patients undergoing CPB, can serve as predictors of postoperative complications and long-term ventricular remodeling [20,21]. Recent evidence suggests that patients with HFrEF have worse postoperative outcomes and show molecular and cellular events associated with greater myocardial inflammation [22,23]. In this context, the NLRP3 inflammasome, which is expressed in cardiomyocytes and cardiac fibroblasts, has been identified as playing a key role in the development of HF and AF, making patients with pre-existing HFrEF the focus of numerous clinical studies exploring intervention strategies at this level. Conversely, HFpEF has been linked to metabolic and non-ischemic risk factors, but further research is needed to better characterize the local inflammation and myocardial remodeling events associated with HFpEF [24].

However, the association of a prooxidant imbalance and inflammation in cardiac tissue in patients with a potentially active remodeling process, such as those with a HFrEF who are undergoing CPB, has not yet been characterized. This pilot clinical study aims to determine if preoperative HFrEF is a factor that influences a prooxidant imbalance in patients subjected to cardiac surgery with CPB and whether this could be related to the activation of the NLRP3 pathway, leading to a higher risk of pAF and, eventually, atrial remodeling phenomena.

## 2. Materials and Methods

### 2.1. Subjects and Clinical Follow-Up

**Figure 1:**
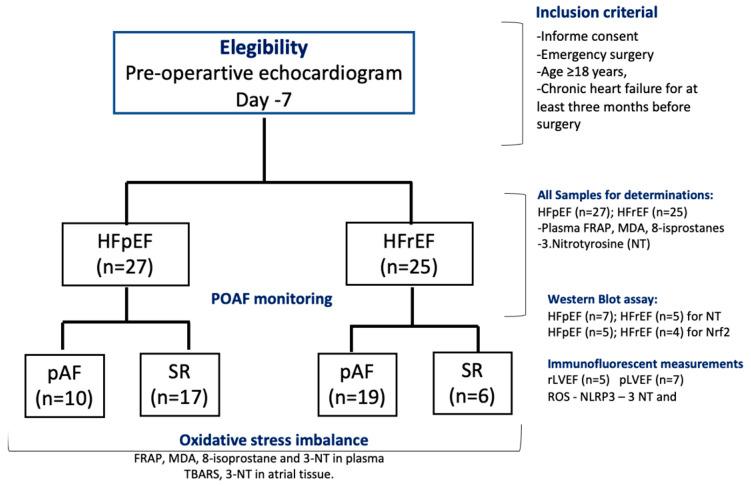
Flow chart of patients’ follow-up, according to CONSORT 2010 guidelines. Included subjects analyzed for each biochemical and WB assay.

A prospective study was conducted on patients referred to the Cardiac Surgery Department at the National Thoracic Institute in Santiago, Chile, from 1 October to 31 December 2023. Fifty-two patients were eligible for the study, which included two groups: (i) HF patients with preserved LVEF (HFpEF, n = 27) and (ii) HF patients with reduced LVEF (HFrEF, n = 25) (Figure 1, CONSORT flow chart). The inclusion criteria were age ≥ 18 years and chronic heart failure (WHO-functional class II, III) for at least three months before surgery. At the baseline visit, 7–10 days before surgery, the patients were classified by echocardiography as HF with pLVEF (HFpEF > 50%) or HF with rLVEF (LVEF ≤ 40%). Patients with a history or evidence of AF, previous myocardial infarction, current use of amiodarone, severe congestive heart failure (New York Heart Association class IV), presence of prosthetic valves, congenital valvular disease, chronic rheumatic, neoplastic diseases, liver insufficiency, severe chronic kidney disease (serum creatinine > 2.5 mg/dL), recent infections (≤2 weeks), and emergency surgery or repair of cyanotic heart disease were excluded from the study. In addition, patients receiving nonsteroidal anti-inflammatory drugs, corticosteroids, antioxidants, vitamins, or fish oil supplements three months before surgery were also excluded. This protocol adhered to the Helsinki standards and was approved by the Local Ethics Committee (06/2020-Bioethic East Health Committee-12/21). The study was registered under ClinicalTrials.gov Identifier NCT06256965.

### 2.2. Post-Operative AF Detection

Continuous electrocardiogram monitoring was performed for 72 h after cardiopulmonary bypass. If arrhythmia symptoms were observed, a 12-lead ECG was performed every 12 h for seven days. The presence of ECG-documented atrial fibrillation lasting at least one minute was classified as a postoperative atrial fibrillation event.

### 2.3. Samples and Biopsies

All patients underwent the same surgical procedure, including standardized induction and anesthesia protocols, with the surgical intervention performed by the same medical team. Surgical access was obtained through a median sternotomy incision, and all anastomoses were sutured by hand. Protection of myocardial tissue was accomplished with crystalloid cold potassium cardioplegic solution. During cardiac surgery, at the time of pericardiocentesis, samples from the right atrial appendage (RA) (approximately 200 mg) and pericardial fluid 10 mL were collected immediately before the initiation of extracorporeal circulation. Biochemical analyses of oxidative stress markers in plasma, atrial tissue, and pericardial fluid (inflammasome NLRP3 pathway), were determined in all recollected samples (n = 25 for HFrEF; n = 27 HFpEF). These samples were immediately frozen in liquid nitrogen and stored at −80 °C. Blood samples were collected in chilled vacutainers containing four mM disodium EDTA and centrifuged at 3000× *g* for 10 min. Plasma samples from each patient were stored at −80 °C until we performed biochemical determinations of NT-proBNP and troponin levels using ELISA assay [25].

### 2.4. Pre-Operative Echocardiographic Images

All echocardiographic analyses were conducted at the National Thorax Institute Echocardiography Unit using GE Vivid E9 equipment at the baseline visit (7 days before surgery). The strain analyses were performed using a semi-automated speckle tracking technique (EchoPAC, EchoPAC Software Only and EchoPAC Plug-in GE Medical Systems, Milwaukee, WI, USA) using a model of the entire LV (based on three apical views). Segments with inadequate tracking were excluded. A 3D full-volume acquisition of the LV was attempted in all patients using a matrix array transducer with the highest possible volume rate. LV volumes and LVEF were measured offline (3DLVQ, EchoPAC, GE Medical Systems, Milwaukee, WI, USA), with abnormal LVEF identified as <40% [26].

### 2.5. Biochemical Parameters

Determinations in plasma, atrial tissue, and pericardial fluid samples (obtained during cardiac surgery at the time of pericardiocentesis) were treated under the same experimental conditions. Regarding these assays were assessed in the same number of samples.

#### 2.5.1. Determination of Oxidative Stress-Related Markers

##### Antioxidant Status

Plasma antioxidant status was assessed by determining the ferric-reducing ability of plasma (FRAP) with a detection limit of 10 μM-Fe^2+^ [27]. FRAP’s inter-assay and intra-assay coefficients of variation (CVs) were 3.0% and 1.0%, respectively. FRAP was expressed as μmol of Fe+2/L of plasma. 

##### Oxidative Stress Markers

Plasma and atrial tissue lipid peroxidation were assessed by the TBARS at pH 3.5, followed by solvent extraction with a mixture of n-butanol/pyridine (15:1, *v*/*v*) [28]. Tetramethoxypropane was used as the external standard, and the levels of lipid peroxides were detected spectrophotometrically at 532 nm and were expressed as μmol malondialdehyde (MDA)/L plasma (umol/L) or mg of protein (Bradford Assay). The inter-assay and intra-assay CVs for TBARS were 10.5% and 4.8%, respectively. In addition, 3-nitrotyrosine (3-NT) in plasma and atrial tissue (Abcam Laboratories, Cambridge, UK, ab116691), and 8-isoprostanes (Cayman Chemicals, Ann Arbor, MI, USA, Item No 516351) were determined with a specific enzyme immunoassay kit following the manufacturer’s recommendations. The ELISA kit’s detection limit for 3-NT was 8 ng/mL, and 8-isoprostanes was 3 pg/mL. The estimated variability of the method for 3-NT was 6.9% for the inter-assay, 13% for the intra-assay, and that of the 8-isoprostanes was 4.2% for the inter-assay, 8.1% for the intra-assay [29,30].

#### 2.5.2. Immunoblot Analysis

The total tissue lysates were stored at −80 °C. The protein contents in the homogenate were measured using the Bradford assay (Bio-Rad, Hercules, CA, USA, 500-0006). Equivalent amounts (30 μg) of each protein extract were denatured in 5× sample buffer (2% sodium dodecyl sulfate, 62.5 mM Tris (pH 6.8), 0.01% bromophenol blue, 1.43 mM mercaptoethanol, and 0.1% glycerol), separated on 10% polyacrylamide gels, and electrophoretically transferred onto a nitrocellulose membrane (PerkinElmer, Providencia, Chile, Protan BA85). After blocking with 4% BSA in 1X PBS, membranes were incubated overnight at 4 °C degree specific antibodies of 3-NT (1:1000; ab61392, Abcam, Cambridge, UK); NRF2 (1:2000; ab31163, Abcam) and the housekeeper proteins, glyceraldehyde-3-phosphate dehydrogenase (Gadph; 1:2000; D16H11, Cell Signaling Technology, Danvers, MA, USA) or α/β-Tubulin (1:2000; #2148; Cell Signaling Technology), followed by goat anti-mouse (1:2000; 31430, Thermo Scientific, Waltham, MA, USA) and rabbit (1:2000; 31460, Thermo Scientific) secondary antibodies, respectively. Immunostaining was performed using chemiluminescent reagents (SuperSignal West Pico Luminol/Enhancer solution; Thermo Scientific, 34080). The immunoblot signals were revealed with a chemiluminescence scanner (Odyssey Imaging System, Li-Cor Biosciences, Lincoln, NE, USA), quantified by densitometry with ScnImage Software, v4.0 (https://scion-image.software.informer.com/4.0/, accessed on 13 January 2025) and normalized by GADPH or α/β-Tubulin protein expression.

#### 2.5.3. RT-PCR NLRP3, IL 1-β and IL-18

According to the manufacturer’s protocol, total RNA was obtained from a rat’s heart employing the SensiFAST cDNA Synthesis kit (Bioline, Toronto, ON, USA). The concentration and purity of RNA were determined by absorbance at 260/280 nm. A Techne Tc-4000 thermal cycler (Thermo Fisher Scientific Inc, Waltham, MA, USA) performed the reverse transcription reaction following the following protocol: 10 min at 25 °C, 15 min at 42 °C, and 5 min at 85 °C. Real-time PCR was performed using Stratagene Mx3000P (Stratagene, La Jolla, CA, USA) using Brilliant III Ultra-Fast SYBR QPCR master mix amplification kit (Agilent Technologies, Santa Clara, CA, USA). The primers used were: NLRP3 Reverse 5′AAC CAA TGC GAG ATC CTG AC 3′/Forward 5′ TGA AGC ATC TGC TCT GAC AC 3′; IL 1 Beta Reverse 5′ TTAGAACCAAATGTGGCCGTG 3′/Forward 5′ TCCCCAGCCCTTTTGTTGA 3’; IL-18 Reverse 5′CTAGAGCGCAATGGTGCAATC3′/Forward5′GACGCATGCCCTCAATCC3′ [31].

A typical reaction contained 250 nmol/L of forward and reverse primer, 1 uL cDNA, and the final reaction volume was 20 uL. All primers used presented optimal amplification efficiency (between 90% and 110%). PCR amplification of the housekeeping gene α-actin was performed as a control. Thermocycling conditions were as follows: 95 °C for 5 min and 40 cycles of 90 °C for 15 s, 60 °C for 15 s, 72 °C for 15 s. Expression values were normalized to α-actin and are reported in units of 2-C+ s.d. as described [32]. CT value was determined by MXPro software (https://www.selectscience.net/product/mxpro-et-qpcr-software, accessed on 13 January 2025) ET when fluorescence was 25% higher than the background. PCR products were verified by melting curve analysis.

#### 2.5.4. Enzyme-Linked Immunosorbent Assay (ELISA)

IL-1β and IL-18 levels in pericardial fluid were analyzed using an ELISA kit according to the manufacturer’s recommendations (Human IL-1 beta ELISA Kit, Catalog Number: ELH-IL1b-Thermo Fisher Scientific Inc., Waltham, MA, USA; Human Total IL-18/IL-1F4, Catalog Number DL180, Thermo Fisher Scientific Inc., Waltham, MA, USA). Briefly, 100 μL of IL-1β and IL-18 standards or cell-free medium of samples were added to human anti-IL-1β and anti-IL-18 antibody-precoated microwells. The microtiter plate was then incubated for two hours at room temperature (RT) and washed with PBS containing 0.5% Tween 20. Afterward, biotinylated anti-IL-1β, anti-IL-18 antibodies, and horseradish peroxidase (HRP)–streptavidin were added to the wells for 1 h at room temperature. In the last step, a substrate solution was added in the dark at RT for 30 min, and the enzymatic reaction was stopped using a stop solution provided with the kit. The absorbances of the immune complexes formed in the wells were read using a TECAN Infinite 200 microplate reader (LifeSciences, Grödig, Austria) at OD450 nm with a reference reading at OD650 nm. Data were calculated according to the standard curves generated by the reference standards of IL-1β and IL-18 and reported as an average of three technical repeats.

#### 2.5.5. proBNP and Troponin Levels in Plasma Samples

proBNP was measured in all EDTA-plasma samples from patients using commercial proBNP Kit assays (Human proBNP ELISA Kit Catalog Number #: ELH-proBNP, RayBiotech, GA, USA) and was expressed as ng/mL. Also, Human Cardiac Troponin I (cTnl) ELISA Kit Catalog #: ELH-CTNI was measured and expressed as pg/mL [33].

#### 2.5.6. Immunolabeling and Fluorescent Microscopy

The tissue samples were mounted in Tissue-Tek, and cryosections 10 μm thick were prepared. Cryo-sections were air dried and fixed for 10 min in 4% paraformaldehyde. After washing in phosphate-buffered saline, sections were incubated with 1% bovine serum albumin for 30 min to block non-specific binding sites. Then, the samples were incubated overnight with primary antibodies against nitrotyrosine (Merck Millipore, Seoul, Republic of Korea, AB 06-264) and NLRP3 (Abcam, ab283819). Secondary antibody was a donkey anti-rabbit IgG-conjugated with Alexa488 (Molecular Probes, Eugene, Oregon). In situ reactive oxygen species (ROS) were determined and quantified using labeling with dihydroethidium as described (see references). Tissue sections were examined by laser scanning confocal microscopy (Leica, Wetzlar, Germany, TCS SP2) or with a fluorescent microscope (Leitz DMRB using a Leica Planapo x40/1.00 or x63/1.32 objective lens, Leica, Wetzlar, Germany).

##### Quantitative Immunofluorescent Measurements

Cryosections from at least two different tissue blocks in each case were used. All samples were immunolabeled simultaneously under identical conditions of fixation and dilutions of primary and secondary antibodies. Sections exposed to PBS instead of primary antibodies served as negative controls. For each patient, at least 10 random fields of vision were analyzed with a fluorescent microscope Leica (Leitz DMRB) using a x40 Planapo objective (Leica). Immunolabeled cryosections were studied using image analysis (Leica) and Image J software (https://imagej.net/ij/download.html, accessed on 10 January 2025). For each protein, a specific setting was established and kept constant in all measurements. The area of specific labeling for ROS, nitrotyrosine, and NLRP3 was calculated as a percentage of positive labeling per 1 square mm of atrial tissue area. Series of confocal optical sections was taken using a Leica Planapo x40/1.00 or x63/1.32 objective lens. Each recorded image was taken using dual-channel scanning and consisted of 1024 × 1024 pixels. To improve image quality and to obtain a high signal-to-noise ratio, each image from the series was signal-averaged. Tissue sections were examined by laser scanning confocal microscopy (Leica TCS SP5). Series of confocal optical sections was taken using a Leica Planapo x63/1.32 objective lens. Each recorded image was taken using multi-channel scanning and consisted of 1024 × 1024 pixels. To improve image quality and to obtain a high signal-to-noise ratio, each image from the series was signal-averaged and was deconvoluted using AutoQuant X2 (Bitplane, Zürich, Switzerland) software. For three-dimensional image reconstructions, an Imaris 6.3.1 multichannel image processing software (Bitplane, Zürich, Switzerland) was used [34,35,36].

For quantification of nitrotyrosine and NLRP3, all tissue samples were immunolabeled simultaneously with identical conditions of fixation and dilutions of primary and secondary antibodies. Ten random fields of vision were quantified using the three-dimensional “Quantification” option of the Imaris program. For each quantification procedure, a specific setting was established and kept constant in all measurements. Quantification of ROS and nitrotyrosine was performed by measuring the fluorescence intensity using a range of 0 to 255 gray values. The quantity of ROS, nitrotyrosine, and NLRP3 was calculated as fluorescent arbitrary units (AU per unit myocardial area (AU/μm^2^) [37].

#### 2.5.7. Statistical Analysis

Shapiro–Wilk test was used to assess whether the data were normally distributed. A two-sample Wilcoxon rank-sum test will be used to evaluate variable medians. If the values are not normally distributed, a U-test and the Kruskal–Wallis statistic will be used. Descriptive statistics of continuous variables were presented as mean ± SEM, or frequency (%), and compared by the Mann–Whitney U test. Spearman test was used to assess the association between oxidative stress markers and LVEF values. A *p*-value < 0.05 was considered statistically significant. Regarding sample size, considering differences in MDA of 40% between groups (HFrEF vs. HFpEF), an SD of 0.5, an effect size of 0.5 for the intervention with 80% power and alpha error of 0.05, 23 patients are needed. (http://www.winepi.net/f108.php, accessed on 4 March 2025). These changes for MDA levels in plasma were analyzed in previous paper published by our group [12].

All statistical analyses were performed using Microsoft Excel and STATA 10.00 for Windows.

## 3. Results

### 3.1. Clinical and Perioperative Characteristics

The mean LVEF was 55.7 ± 8.4 for the HFpEF group and 35.3 ± 7.1 for the HFrEF group (*p* = 0.02). Baseline clinical characteristics were obtained 7 days before surgery (Table 1). There were no significant differences in the demographic characteristics, comorbidities, and preoperative pharmacotherapy between the two groups. Specifically, in terms of preoperative pharmacotherapy, the study noted that the use of beta-blockers and angiotensin receptor blockers, which could influence the ventricular contractile response and AF incidence, was similar across both groups. In terms of echocardiographic parameters, the HFrEF group showed higher LA volume index (17.4 *±* 2.4 vs. 12.5 *±* 1.5, *p* = 0.04) and LV mass index (116.3 *±* 7.8 vs. 87.5 *±* 2.5, *p* = 0.03), indicating LV diastolic dysfunction and LV hypertrophy, respectively. Regarding perioperative features, there were no significant differences in hemodynamic variables associated with cardiopulmonary bypass.

### 3.2. Antioxidants and Oxidative Stress Markers in Plasma and Atrial Tissue

**Figure 2:**
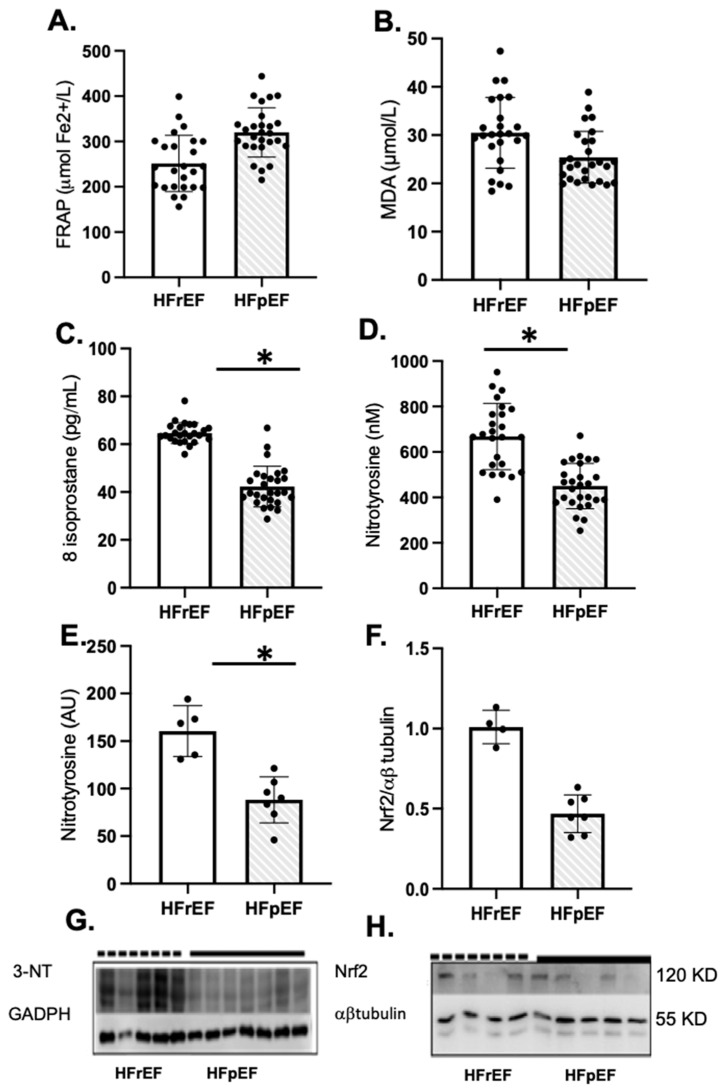
Oxidative stress and antioxidant status of enrolled patients preoperatively as pLVEF (n = 27) or rLVEF (n = 25). Plasma antioxidant capacity (**A**), FRAP, the ferric reducing ability of plasma, malondialdehyde (**B**), and 8-isoprostane levels in plasma (**C**). Nitrotyrosine levels in plasma (**D**) and atrial tissue (**E**) and NRF2 protein levels in atrial tissue (**F**). (**G**,**H**) groups are photographs representative of Western blot 3-NT for HFpEF (n = 7) and HFrEF (n = 5); and NRF2 for HFpEF (n = 5) and HFrEF (n = 4). Values are means ± SEM. Significant differences (*p* ≤ 0.05): * vs. pLVEF. FRAP: ferric reducing ability of plasma; MDA: malondialdehyde; NT, nitrotyrosine; GADPH, glyceraldehyde-3-phosphate dehydrogenase.

Patients with HFrEF showed a significant increase in oxidative markers of lipid peroxidation and protein oxidation, such as 8-isoprostane and nitrotyrosine levels in plasma and atrial tissue, compared to HFpEF patients, respectively (Figure 2C–E). However, Nrf2 protein levels were similar in both groups (Figure 2F). The full-screen western blot are included in Supplementary Figure S1.

### 3.3. Association Between LVEF and Oxidative Stress Markers

**Figure 3:**
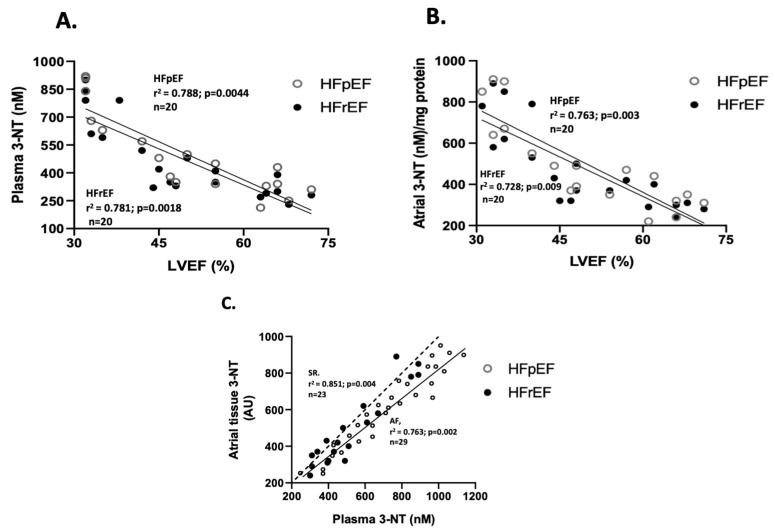
Association between LVEF and oxidative stress markers. Correlation between values of LVEF (%) and plasma nitrotyrosine levels (nmoles/L) (**A**) and atrial tissue levels (arbitrary units, AU) (**B**). Correlation between atrial and blood samples of 3-NT at surgical time (**C**). The Spearman test obtained values of r^2^.

Regarding the correlation between oxidative stress marker values and LVEF (%), only nitrotyrosine levels showed a negative correlation in both groups. Specifically, for 3-NT plasma levels (n = 20), the correlation coefficients were r^2^= 0.781 (*p* = 0.0018) in the HFrEF group, and r^2^= 0.788 (*p* = 0.0044) in the HFpEF group (Figure 3A). Similarly, for 3-NT atrial tissue levels (n = 20), r^2^= 0.728 (*p* = 0.009) in the HFrEF, and r^2^= 0.763 (*p* = 0.003) in the HFpEF group (Figure 3B). Additionally, a positive correlation between 3-NT levels at surgical time was observed between atrial and blood samples (n = 20), with r^2^ = 0.893 (*p* = 0.002) (Figure 3C).

### 3.4. NLRP3 Inflammasome in Atrial Tissue and Pericardial Fluid

**Figure 4:**
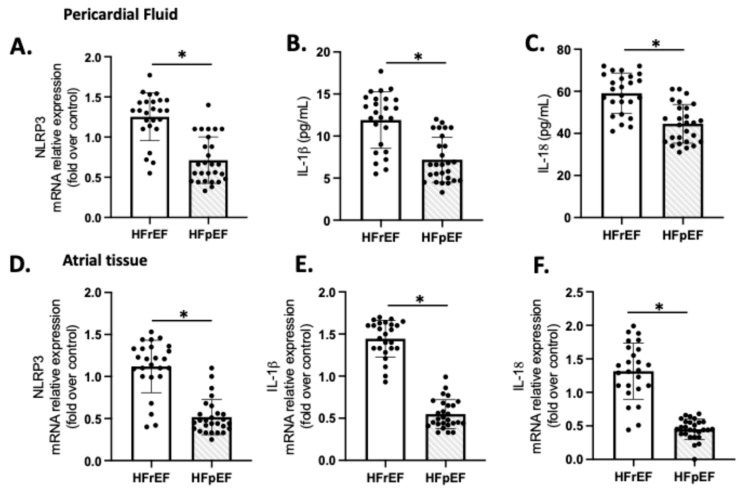
Atrial tissue (**A**–**C**) and pericardial fluid (**D**–**F**) expression of NLRP3, IL-1β, and IL-18. Values are means ± SEM for pLVEF (n = 27) and rLVEF (n = 25). The black circles are the number of samples of each group. Significant differences (*p* ≤ 0.05): * vs. HFpEF.

We examined the expression of NLRP3, IL-1β, and IL-18 as indicators of sterile inflammation in 20 samples of patients with preoperative HFrEF and HFpEF who were subjected to CPB (Figure 4). The mRNA levels of NLRP3, IL-1β, and IL-18 in the HFrEF group were significantly higher compared to the respective values in the pericardial fluid (Figure 4A–C) and atrial tissue (Figure 4D–F) of the HFpEF group (*p* < 0.01).

### 3.5. Atrial Fibrillation and Oxidative Stress Markers

**Figure 5:**
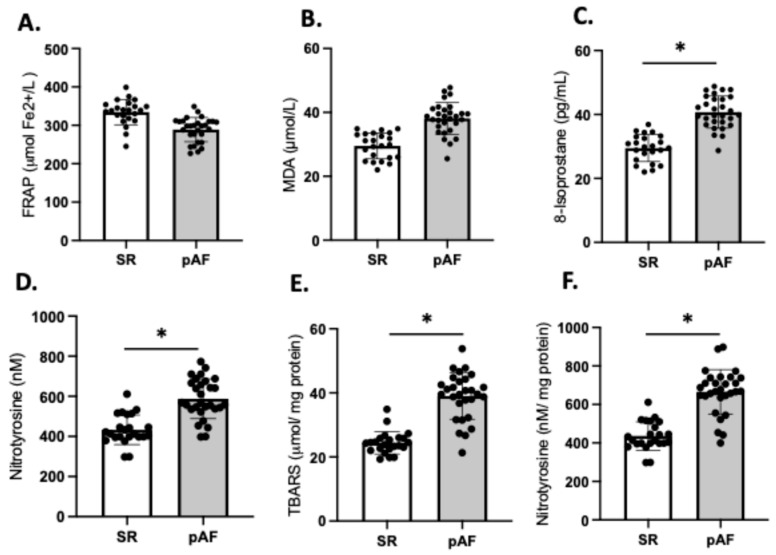
Redox imbalance of patients who developed atrial fibrillation (AF) (n = 29) and who maintained sinus rhythm (SR) (n = 23). Plasma antioxidant capacity; (**A**) FRAP, the ferric reducing ability of plasma. (**B**) MDA, malondialdehyde in plasma, (**C**) 8-isoprostane levels in plasma, (**D**) nitrotyrosine in plasma. (**E**) TBARS in atrial tissue, (**F**) 3 NT (nitrotyrosine) in atrial tissue. The black circles are the number of samples of each group. Values are means ± SEM. Significant differences (*p* ≤ 0.05): * vs. sinus rhythm (SR).

We compared the redox balance of patients who developed postoperative AF (n = 29) (55%) with those patients who maintained sinus rhythm (SR) (n = 23); the frequencies align with values reported in the literature [12,14]. Our analysis revealed no significant differences in FRAP and MDA between the two groups (Figure 5A,B). However, the plasma levels of 8-isoprostane were significantly higher in AF patients compared to those in SR (SR: 29.6 ± 4.7 vs. AF: 41.7 ± 7.3; *p* = 0.03), as were the levels of 3-NT (SR: 422 ± 97 vs. AF: 657 ± 112; *p* = 0.03) (Figure 5C,D). These findings were consistent with higher levels of TBARS (40.8 ± 7.2 vs. 22.7 ± 1.9; *p* = 0.02) (Figure 5E) and 3-NT (706.1 ± 33 vs. 481.7 ± 27; *p* = 0.03) (Figure 5F) in atrial tissue of patients who developed AF compared to those who maintained SR.

### 3.6. Immunohistochemistry and Quantification of ROS, Nitrotyrosine, and NLRP3 in Atrial Biopsies

**Figure 6:**
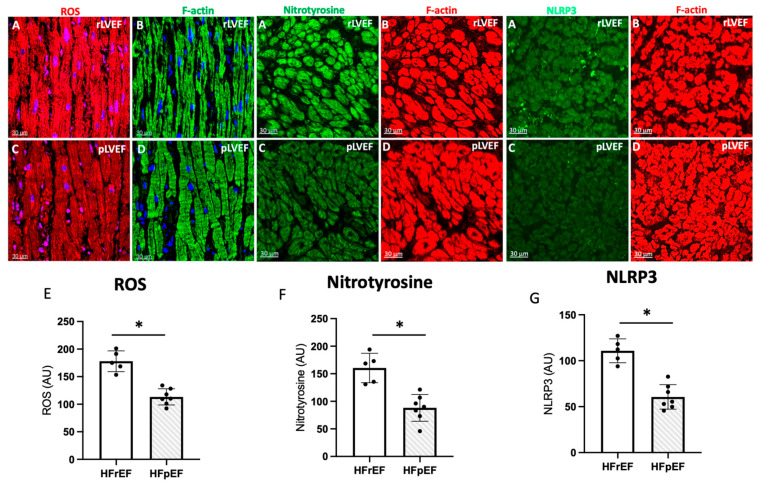
Representative photo of ROS (red), nitrotyrosine (green) and NLRP3 (green) (**A**), and respectively quantification (**E**–**G**) of images in atrial biopsies from HFrEF (rLVEF n = 5) and HFpEF (pLVEF, n = 7) patients with develop pAF (**C**) represented ROS, NT and NLRP3 in pLVEF group; F-actin was labeled either green (**B**) (rLVEF) or red (**D**) (pLVEF). Blue was used to stain the nuclei with DAPI. The graphs represent the quantification of ROS, nitrotyrosine, and NLRP3. Black circles are the samples of each group. Scale bar (30 μm) was added in each image. Significant differences (*p* ≤ 0.05): * vs. HFrEF.

Next, we have employed confocal microscopy and quantitative immunohistochemistry to study ROS. Representative confocal images of atrial tissue sections from pAF patients with rLVEF (Figure 6A,B) and with pLVEF (Figure 6C,D). Dihydroethidium staining for ROS is shown in red color, left panels. Cardiomyocytes are stained green with F-actin (right panels). Note that the ROS, nitrotyrosine, and NLRP3 signals in the atrial tissue of patients with rLVEF are significantly increased compared to patients with pLVEF.

## 4. Discussion

In this study, we found that preoperatively, HFrEF is a clinical factor associated with a higher systemic and cardiac tissue prooxidant status compared with HFpEF, in patients undergoing cardiac surgery with CPB. Our approach, which utilizes biochemical markers in plasma, pericardial fluid, and atrial samples, provides a molecular perspective that complements the functional findings obtained from echocardiography. Our findings suggest that oxidative stress and sterile inflammation occurring locally in both groups could be a mechanism associated with myocardial injury in patients who develop AF.

The association between postoperative AF and oxidative stress has been well documented from both a clinical and mechanistic point of view [12,38]. The technical procedure applied in cardiac surgeries can injure myocardial tissue primarily due to changes in perfusion and oxygenation, leading to the formation of ROS [14,39]. Several mechanisms, such as mitochondrial respiration and neutrophil activation, can generate ROS during cardiopulmonary bypass [40]. The production of ROS during the early reperfusion phase, combined with the decrease in antioxidant defenses induced by the IR cycle, makes myocardial tissue extremely vulnerable to oxidative damage [41]. Among these, key species involved in this mechanism of damage include the superoxide radical (^•^O_2_^−^), hydroxyl radical (^•^OH^−^), and peroxynitrite (NOO^−^). These species have been shown to cause damage in various experimental models and individuals subjected to postinfarction thrombolysis and stroke [42], percutaneous angioplasty [43], and cardiothoracic surgery [44]. Because cell membranes are primarily composed of phospholipids and proteins, redox modifications in these compounds by ROS are essential factors in the induction and consequences of atrial tissue damage. For instance, lipoperoxidation and the loss of membrane integrity may lead to a drop in ATP levels and an overload of cytosolic calcium, cellular events that contribute to cell death and contractile dysfunction [45,46]. In addition, ROS can act as mediators or messengers, triggering intracellular signals that activate transcription factors such as NF-κB, leading to the expression of pro-inflammatory genes [47]. Once the inflammatory process is initiated, the leukocytes’ transmigration and activation occur, enhancing local oxidative stress [48,49]. The release of mediators such as cytokines, chemokines, and adhesion molecules may exacerbate the tissue damage, resulting in focal myocardial necrosis [9]. Tissue repair involves the risk of collagen deposition in the extracellular matrix, which can lead to interstitial fibrosis and affect both electrical and mechanical properties through remodeling [50]. Thus, increases in ROS concentration can affect the contractile function of cardiomyocytes associated with calcium overload and enhance the sensitivity of myofilaments as an arrhythmogenic mechanism [51].

Our data demonstrated high levels of oxidative stress-related modifications in proteins, such as nitrotyrosine residues in plasma and atrial tissue proteins from patients with preoperative rLVEF (Figure 2D,E), and those who developed postoperative AF (Figure 4D–F). We also observed elevated plasma levels of 8-isoprostanes, as an in vivo lipid peroxidation marker in perioperative samples (Figure 2C). In some animal models of pressure overload, a relationship has been described between LV function and oxidative stress levels in cardiac tissue [52]. In addition, a model of diabetic cardiomyopathy showed similar ventricular pathological features in hypertrophic cardiomyocytes as those seen in a volume overload model, illustrating similarities in terms of oxidative effects. Both models exhibited higher levels of nitrotyrosine in the myocardium of rats [53,54]. Nitrosative stress plays an important role in the progression of chronic heart failure [54]. Similar to ROS, reactive nitrogen species (RNS) leads to myocyte apoptosis, direct negative inotropic effects, and reduced bioavailability of nitric oxide (NO). RNS can cause vasoconstriction in the coronary, pulmonary, and peripheral vasculature; however, the acute effects on the myocardium have not been well characterized. Also, the amount of oxidative damage caused by RNS in the LV could be similar to that seen in animal models of cardiac IR, which is characterized by a ROS burst [55,56,57]. Some mechanisms involved in this injury include the induction of mitochondrial dysfunction and cell death by apoptosis pathways. Regarding pre-operative factors and oxidative stress occurrence, cardiometabolic risk factors and hemodynamic state could influence the pro-oxidant sources (e.g., myeloperoxidase) in leukocytes and myocardial tissue, similar to IR injury [58].

While most clinical studies and animal models focus on the mechanisms related to IR injury following cardiac surgery [41,59,60], few studies have investigated the impact of lower preoperative cardiac function or ventricular remodeling. These factors can be influenced by the patient’s comorbidities or the degree of structural heart disease before cardiac surgery [61,62]. For example, research indicates that patients with HFpEF, even in the absence of cardiovascular signs or symptoms of HF, may exhibit structural changes in cardiomyocyte morphology and contractile properties similar to those seen in patients with HFrEF, but with only minimally elevated plasma biomarkers of cardiac injury [63]. In this view, our findings demonstrate a negative correlation between 3-NT levels in plasma and atrial tissue with a decrease in LV function across both patient groups (Figure 3A,B). Additionally, we observed a direct relationship between 3-NT levels in atrial and surgical blood samples (Figure 3C). Previous studies aimed at testing antioxidant supplementation before cardiac surgery, found higher 3-NT levels in the atrial tissue of patients who received placebo treatment and experienced post-operative atrial fibrillation (pAF). In contrast, those who received combined therapy with omega-3 and antioxidant vitamins exhibited lower 3-NT levels [64]. Furthermore, some protocols analyzing human samples from patients with valvular disease collected prior to cannulation of the right atrial appendage indicated significantly elevated 3-nitrotyrosine levels in this clinical context [65]. Therefore, it is crucial to identify additional markers with mechanistic implications to detect those clinical cases with a higher risk of progression to structural damage due to pathological remodeling in patients undergoing CPB.

The role of oxidative stress and inflammation in the postoperative outcomes could be determined by baseline cardiac function or structural remodeling. Pre-existing cardiovascular risk factors and technical procedures during surgery can also lead to direct pro-inflammatory injury to cardiac tissue [41,66]. For example, in patients with pre-existing cardiac conditions who undergo cardiac bypass or valve replacement surgeries (for stenosis or severe valvular insufficiency), the extent of left ventricular remodeling or dilation may contribute to postoperative complications. Abnormalities in myocardial compliance and deformation, such as alterations in global longitudinal strain, have been associated with the development of AF following isolated cardiac surgery [67]. Preoperative LA strain has been linked to the onset of postoperative AF following isolated coronary artery bypass surgery [22]. Moreover, decreased LV strain rates and wall stress/LV volume index following mitral valve repair may determine a contractile dysfunction, even if the pre-surgical LVEF is above 60% [68]. In this setting, the main factor could be oxidative stress occurrence, which could cause myofibrillar degeneration [69].

The inflammasome is a crucial component of innate immunity and plays a role in various pathophysiologic processes. Among them, the NLRP3 inflammasome has been the most extensively studied, recognizing multiple pathogens through pattern recognition receptors of the innate immunity system and mediating inflammatory responses via Caspase-1 activation [11]. Both preclinical and clinical findings support the significant role of the NLRP3 inflammasome and IL-1 cytokines in the formation, progression, and complications of atherosclerosis, in ischemic injury (such as acute myocardial infarction), and in non-ischemic injury to the myocardium (myocarditis), leading to heart failure [70]. Consequently, clinically available IL-1 inhibitors, as well as NLRP3 inflammasome inhibitors currently in clinical development, are of particular interest [71]. Canakinumab, an IL-1β antibody, has been shown to prevent the recurrence of ischemic events in patients with prior acute myocardial infarction in a large phase III clinical trial, involving 10.061 participants worldwide [72], Ridker et al., 2017. Phase II clinical trials have also yielded promising results for anakinra, a recombinant IL-1 receptor antagonist, in patients with ST-segment-elevation acute myocardial infarction or HFrEF [73]. Also, several studies suggest that NLRP3 inflammasome activation contributes to the onset and development of AF [74,75].

Concerning atrial detection of ROS and NLRP3, increased oxidative and proinflammatory damage may be the mechanistic connection between acute damage events, the substrate for the occurrence of arrhythmias such as pAF, and the eventual triggers of ventricular remodeling [10,76]. In this issue, our results show that structurally, patients with HFrEF who develop pAF have higher detection of ROS, nitrotyrosine, and NLRP3 (Figure 6); therefore, molecular pathways that favor ventricular remodeling in these patients.

While some research has highlighted long-term effects such as ventricular remodeling events [77,78], the short-term effects related to clinical injury due to IR, such as those seen in CPB, are still underexplored. Regarding our protocol, higher levels of NLRP3, IL-1beta, and IL-18 expression were shown in both pericardial fluid and atrial tissue of patients with HFrEF compared to HFpEF (Figure 4). These data could reveal a certain pathogenetic role in the occurrence of ventricular failure or a mechanism triggered by the IR cycle and local ROS burst [10,79]. There is also significant interest in exploring new therapeutic paradigms that focus on this type of sterile, low-grade inflammation that conditions the progression of heart failure.

Our pilot study results suggest that oxidative stress may contribute to the dynamics of ventricular remodeling in patients with HFrEF and HFpEF. These findings are consistent with the proposals of some clinical studies, where antioxidants were administered to HFrEF patients, resulting in improved LV function [80,81]. At a molecular level, there is a connection between antioxidant responses and certain redox-sensitive transcription factors linked to contractile dysfunction [82]. For instance, the nuclear factor erythroid 2-related factor 2 (Nrf2) serves as a transcriptional regulator known to provide transient cytoprotection to the myocardium following acute ischemic insults. However, the sustained activation of Nrf2 may paradoxically cause a reductive environment characterized by excessive antioxidant activity and increased reduced glutathione (GSH) levels [83]. The circulatory redox state, indicated by the GSH/MDA ratio, has been used to categorize HF patients into normal redox or hyper-oxidative (HO) groups. Among HF-HO patients, the levels of antioxidant enzyme proteins, such as superoxide dismutase, glutathione peroxidase, and catalase, are significantly elevated, while glutathione reductase activity is notably reduced [84]. Furthermore, reductions in GSH and ascorbate levels have been associated with impaired calcium loading in the cytosol in some models of AF [85,86]. Moreover, ascorbate supplementation can reduce peroxynitrite-mediated injury and mitigate or eliminate the atrial electrophysiological remodeling linked to these pathophysiological processes [87,88]. Therefore, these findings support the Nrf2/antioxidant response elements (ARE) pathway as a potential contributor to cardiac antioxidant status and provide novel candidates for future mechanistic investigations to better understand the relationship between myocardial OS and the pathophysiology of cardiac IR injury [89,90]. Notably, in our results, the protein levels of total Nrf2 did not exhibit changes in atrial samples from both groups (Figure 2H). This could be partly explained by the slow nature of redox-type transcriptional changes, suggesting that there may not be enough time for an efficient antioxidant response. Evidence of this lack of response is reflected in the absence of changes in plasma antioxidant capacity (Figure 2A).

Finally, the association of functional parameters such as LVEF and LVGS (%) with postoperative outcomes has been the focus of a few short-term studies [91]. In this case, patients with HFrEF, who have greater tissue damage due to OS, should have worse outcomes, such as postoperative AF [12,14]. These changes may be even milder in patients with HFpEF.

The predictive value of ventricular function is crucial for determining the long-term outcomes of patients undergoing CPB, particularly for those with an LVEF below 30% [92]. LVGS values obtained by speckle tracking echocardiography (STE) can more effectively detect subclinical changes in patients after CPB [93]. In patients with previous HFpEF, changes in LVEF can be subtle and may not be easily identified during the acute phase or in the postoperative follow-up after CPB [94]. Furthermore, patients with HFpEF may initially remain asymptomatic despite experiencing subclinical effects on ventricular function, making detection even more challenging [95]. Regarding the timing of LVGS assessment for prognostic value in patients undergoing CPB, some studies suggest that preoperative LVGS values may better predict long-term prognosis [96]. Conversely, there is evidence to suggest that postoperative LVGS may have greater prognostic value than preoperative measurements [97]. Therefore, the optimal timing for LVGS evaluation to enhance its prognostic utility remains unclear, and further prospective studies are needed to define this aspect more precisely.

### Limitations of the Study

Our study had certain limitations. Firstly, we did not conduct a long-term postoperative follow-up to assess the effects over an extended time. Secondly, due to the limited amount of atrial tissue samples available for biochemical analysis, we were unable to analyze additional protein markers using Western blot techniques and in AF patients. However, a previous cross-sectional observational study of coronary patients conducted in the same hospital and surgical unit revealed low inflammatory and remodeling markers in patients with preoperative HFpEF after six months of follow-up [89,90]. These clinical and biochemical findings support the need to explore other markers, such as inflammatory mediators or remodeling indicators.

## 5. Conclusions

Increased oxidative and nitrosative stress and elevated NLRP3 inflammasome levels favor cardiac injury after CPB. Preoperatively, patients with HFrEF showed increased NLRP3 inflammasome expression in atrial and pericardial fluid samples, suggesting a potential pharmacologic target to treat clinical complications due to IR damage. In addition, combining echocardiographic parameters of contractile dysfunction with OS markers could be helpful for identifying the patients who would benefit from preoperative pharmacologic therapy with antioxidants. This approach could be used as a strategy to prevent pAF and other clinical complications related to increased OS in patients with CPB, especially in patients with preoperative HFrEF.

## Figures and Tables

**Table 1:** General clinical characteristics of patients.

Clinical Parameters	rLVEF (n = 25)	pLVEF (n = 27)	*p*-Value
Age	57.6 (56–61)	64.7 (58–67)	0.63
Sex (M/F)	15/10	18/9	0.47
BMI	29.8 (25–33)	31.3 (26–35)	0.45
Comorbidities			
Essential hypertension	12	14	0.66
D. Mellitus	13	15	0.78
Chronic pulmonary disease	12	11	0.88
Hypercholesterolemia	12	12	0.75
Smoking history	11	12	1.00
Pharmacotherapy			
Aspirin	12	14	0.85
Statins	21	21	0.71
Diuretics	1	--	--
Beta-blockers	14	16	0.69
Nitrates	12	11	0.77
ACEI/ARB	14	16	0.78
Sulfonylureas	11	13	0.49
Biguanides	13	14	0.33
Gliflozins	7	8	0.78
M. injury markers			
hs-cTn (ng/L)	13.25 ± 7.4	7.17 ± 2.8	0.46
BNP (ng/mL)	145 ± 27.6	98 ± 16.8	0.77
Echocardiographical parameters			
LVEF (%)	35.3 ± 7.1	55.7 ± 8.4 *	0.04
LVGS (%)	−21%	−18.8	0.66
LA volumen index (mL/m^2^)	17.4 ± 2.3 *	12.5 ± 1.5	0.04
LV end-systolic			
dimension (mm)	30.1 ± 1.8	29.3 ± 1.9	0.07
LV end-diastolic	41.1 ± 1.3	43.7 ± 2.1	0.68
dimension (mm)			
LV mass index (g/m^2^)	116.3 ± 7.8 *	85.7 ± 2.5	0.03
E/A ratio	1.6 ± 0.05	1.4 ± 0.04	0.22
E/e’ ratio	6.0 ± 0.95	6.3 ± 0.77	0.45

Values are median (interquartile range) or n (%). * *p* < 0.05, vs. HFpEF. ACEI = angiotensin-converting enzyme inhibitors; ARB = angiotensin receptor blocker; LV = left ventricular; hs-cTn, high-sensitivity cardiac troponin; BNP, B-type natriuretic peptide; M = myocardial; LVGS, left ventricular global strain.

## Data Availability

Data is contained within the article and Supplementary Materials.

## Supplementary Materials

The following supporting information can be downloaded at: https://www.mdpi.com/article/10.3390/antiox14111311/s1.

## Abbreviations

The following abbreviations are used in this manuscript:

ARE	antioxidants response elements
CPB	cardiopulmonary bypass
HFrEF	heart failure with reduced ejection fraction
HFpEF	heart failure with preserved ejection fraction
FRAP	ferric reducing ability of plasma
GSH	glutathione
IL-1	interleukin-1
IL-1β	interleukin-1 beta
IL-18	interleukin-18
Nrf2	Nuclear factor erythroid 2-related factor 2
NOO-	peroxynitrite
NLRP3	NOD-, LRR- and pyrin domain-containing protein 3
NADPH	nicotinamide adenine dinucleotide phosphate
3-NT	3-Nitrotyrosine
OS	oxidative stress
^•^OH	hydroxyl radical
^•^O_2_^−^	superoxide radical
pAF	postoperative atrial fibrillation
ROS	reactive oxygen species
TBARS	thiobarbituric acid reactive substances

## References

[1] Lazzarin T., Martins D., Ballarin R.S., Monte M.G., Minicucci M.F., Polegato B.F., Zornoff L. (2023). The Role of Omega-3 in Attenuating Cardiac Remodeling and Heart Failure through the Oxidative Stress and Inflammation Pathways. Antioxidants.

[2] Omote K., Verbrugge F.H., Borlaug B.A. (2022). Heart Failure with Preserved Ejection Fraction: Mechanisms and Treatment Strategies. Annu. Rev. Med..

[3] Langlois P.L., Hardy G., Manzanares W. (2017). Omega-3 polyunsaturated fatty acids in cardiac surgery patients: An updated systematic review and meta-analysis. Clin. Nutr..

[4] Chen S., Acou W.J., Kiuchi M.G., Meyer C., Sommer P., Martinek M., Schratter A., Andrea B.R., Ling Z., Liu S. (2017). Association of Preoperative Renin-Angiotensin System Inhibitors with Prevention of Postoperative Atrial Fibrillation and Adverse Events: A Systematic Review and Meta-analysis. JAMA Netw. Open.

[5] Hogue C.W., Creswell L.L., Gutterman D.D., Fleisher L.A. (2005). Epidemiology, mechanisms, and risks: American College of Chest Physicians guidelines for the prevention and management of postoperative atrial fibrillation after cardiac surgery. Chest.

[6] Dobrev D., Aguilar M., Heijman J., Guichard J.-B., Nattel S. (2019). Postoperative atrial fibrillation: Mechanisms, manifestations and management. Nat. Rev. Cardiol..

[7] Goulden C., Hagana A., Ulucay E., Zaman E., Ahmed A., Harky A. (2022). Optimising risk factors for atrial fibrillation post-cardiac surgery. Perfusion.

[8] Andrade J., Khairy P., Dobrev D., Nattel S. (2014). The clinical profile and pathophysiology of atrial fibrillation: Relationships among clinical features, epidemiology, and mechanisms. Circ. Res..

[9] Harada M., Van Wagoner D.R., Nattel S. (2015). Role of inflammation in atrial fibrillation pathophysiology and management. Circ. J..

[10] Castillo R.L., Farías J.G., Sandoval C., González-Candia A., Figueroa E., Quezada M., Cruz G., Llanos P., Jorquera G., Kostin S. (2024). Role of NLRP3 Inflammasome in Heart Failure Patients Undergoing Cardiac Surgery as a Potential Determinant of Postoperative Atrial Fibrillation and Remodeling: Is SGLT2 Cotransporter Inhibition an Alternative for Cardioprotection?. Antioxidants.

[11] Smorodinova N., Bláha M., Melenovský V., Rozsívalová K., Přidal J., Ďurišová M., Pirk J., Kautzner J., Kučera T. (2017). Analysis of immune cell populations in atrial myocardium of patients with atrial fibrillation or sinus rhythm. PLoS ONE.

[12] Rodrigo R., Korantzopoulos P., Cereceda M., Asenjo R., Zamorano J., Villalabeitia E., Baeza C., Aguayo R., Castillo R., Carrasco R. (2013). A randomized controlled trial to prevent postoperative atrial fibrillation by antioxidant reinforcement. J. Am. Coll. Cardiol..

[13] Gnoni A., Ballini A., Trentadue R., Taurin F., Santacroce L., Ferrara P., Massaro F., Brienza N., Massari A.M., Sardaro N. (2019). Induction of mitochondrial dysfunction in patients under cardiopulmonary by-pass: Preliminary results. Eur. Rev. Med. Pharmacol. Sci..

[14] Castillo R.L., Rodrigo R., Pérez F., Cereceda M., Asenjo R., Zamorano J., Navarrete R., Villalabeitia E., Sanz J., Baeza C. (2011). Antioxidant therapy reduces oxidative and inflammatory tissue damage in patients subjected to cardiac surgery with extracorporeal circulation. Basic. Clin. Pharmacol. Toxicol..

[15] Liu C., Huang Z.H., Huang S.H., Jou T. (2021). Endocytosis of peroxiredoxin 1 links sterile inflammation to immunoparalysis in pediatric patients following cardiopulmonary bypass. Redox Biol..

[16] Korantzopoulos P., Letsas K., Fragakis N., Tse G., Liu T. (2018). Oxidative stress and atrial fibrillation: An update. Free Radic. Res..

[17] Sánchez F.J., Gonzalez V.A., Farrando M., Jayat A.O., Segovia-Roldan M., García-Mendívil L., Ordovás L., Prado N.J., Pueyo E., Diez E.R. (2020). Atrial Dyssynchrony Measured by Strain Echocardiography as a Marker of Proarrhythmic Remodeling and Oxidative Stress in Cardiac Surgery Patients. Oxid. Med. Cell Longev..

[18] Colombo P., Castagna F., Onat D., Wong K.Y., Harxhi A., Hayashi Y., Friedman R.A., Pinsino A., Ladanyi L., Mebazaa A. (2024). Experimentally Induced Peripheral Venous Congestion Exacerbates Inflammation, Oxidative Stress, and Neurohormonal and Endothelial Cell Activation in Patients with Systolic Heart Failure. J. Card. Fail..

[19] Mathis M.R., Duggal N.L., Janda J.M., Fennema J.L., Yang B., Pagani F.D., Maile M.D., Hofer R.E., Jewell E.S., Engoren M.C. (2021). Reduced Echocardiographic Inotropy Index after Cardiopulmonary Bypass Is Associated with Complications After Cardiac Surgery: An Institutional Outcomes Study. J. Cardiothorac. Vasc. Anesth..

[20] Nakae M., Kainuma S., Toda K., Miyagawa S., Yoshikawa Y., Hata H., Yoshioka D., Kawamura T., Kawamura A., Kashiyama N. (2021). Incidence, determinants and clinical impact of left ventricular function recovery after surgical treatments for ischaemic cardiomyopathy. Eur. J. Cardiothorac. Surg..

[21] Kislitsina O.N., Cox J.L., Shah L.J., Malaisrie S.C., Kruse J., Liu M., Andrei A., McCarthy P.M. (2020). Preoperative left atrial strain abnormalities are associated with the development of postoperative atrial fibrillation following isolated coronary artery bypass surgery. J. Thorac. Cardiovasc. Surg..

[22] Vasan R.S., Urbina E.M., Jin J., Xanthakis V. (2021). Prognostic Significance of Echocardiographic Measures of Cardiac Remodeling in the Community. Curr. Cardiol. Rep..

[23] van Wezenbeek J., Canada J.M., Ravindra K., Carbone S., Trankle C.R., Kadariya D., Buckley L.F., Del Buono M., Billingsley H., Viscusi M. (2018). Reactive Protein and N-Terminal Pro-brain Natriuretic Peptide Levels Correlate with Impaired Cardiorespiratory Fitness in Patients with Heart Failure Across a Wide Range of Ejection Fraction. Front. Cardiovasc. Med..

[24] Kostin S., Krizanic F., Kelesidis T., Pagonas N. (2024). The role of NETosis in heart failure. Heart Fail. Rev..

[25] Hidayet S., Yağmur Y., Bayramoğlu A., Taşolar M.H., Kurtoğlu E., Özyalın F. (2018). Prediction of postoperative atrial fibrillation with left atrial mechanical functions and NT-pro ANP levels after coronary artery bypass surgery: A three-dimensional echocardiography study. Echocardiography.

[26] Thavendiranathan P., Negishi T., Somerset E., Negishi K., Penicka M., Lemieux J., Aakhus S., Miyazaki S., Shirazi M., Galderisi M. (2021). Strain-Guided Management of Potentially Cardiotoxic Cancer Therapy. J. Am. Coll. Cardiol..

[27] Benzie I.F., Strain J.J. (1996). The ferric reducing ability of plasma (FRAP) as a measure of ‘antioxidant power’: The FRAP assay. Anal. Biochem..

[28] Ohkawa H., Ohishi N., Yagi K. (1979). Assay for lipid peroxides in animal tissues by thiobarbituric acid reaction. Anal. Biochem..

[29] Szymczyk G., Bełtowski J., Marciniak A., Kotarski J. (2005). Serum isoprostanes levels in patients after abdominal hysterectomy. Rocz. Akad. Med. Bialymst..

[30] Gutiérrez-Camacho L.R., Kormanovski A., Castillo-Hernández M., Guevara-Balcázar G., Lara-Padilla E. (2020). Alterations in glutathione, nitric oxide and 3-nitrotyrosine levels following exercise and/or hyperbaric oxygen treatment in mice with diet-induced diabetes. Biomed. Rep..

[31] Manfrere K., Torrealba M.P., Ferreira F.M., Alho de Sousa E.S., Miyashiro D., Teixeira F.M., Custódio R.W., Nakaya H., Ramos Y.A., Sotto M.N. (2023). Imbalanced IL-1B and IL-18 Expression in Sézary Syndrome. Int. J. Mol. Sci..

[32] Pfaffl M.W. (2001). A new mathematical model for relative quantification in real-time RT-PCR. Nucleic Acids Res..

[33] Atas E., Kismet E., Kesik V., Karaoglu B., Aydemir G., Korkmazer N., Demirkaya E., Karslioglu Y., Yurttutan N., Unay B. (2015). Cardiac troponin-I, brain natriuretic peptide and endothelin-1 levels in a rat model of doxorubicin-induced cardiac injury. J. Cancer Res. Ther..

[34] Kostin S., Richter M., Krizanic F., Sasko B., Kelesidis T., Pagonas N. (2025). NETosis Is an Important Component of Chronic Myocardial Inflammation in Patients With Heart Failure. Circ. Heart Fail..

[35] Cabrera-Fuentes H.A., Ruiz-Meana M., Simsekyilmaz S., Kostin S., Inserte J., Saffarzadeh M., Galuska S.P., Vijayan V., Barba I., Barreto G. (2014). RNase1 prevents the damaging interplay between extracellular RNA and tumour necrosis factor-α in cardiac ischaemia/reperfusion injury. Thromb. Haemost..

[36] Abassi Z.A., Barac Y.D., Kostin S., Roguin A., Ovcharenko E., Awad H., Blank A., Bar-Am O., Amit T., Schaper J. (2011). TVP1022 attenuates cardiac remodeling and kidney dysfunction in experimental volume overload-induced congestive heart failure. Circ. Heart Fail..

[37] Bricker-Anthony C., Rex T.S. (2015). Neurodegeneration and Vision Loss after Mild Blunt Trauma in the C57Bl/6 and DBA/2J Mouse. PLoS ONE.

[38] Noubiap J.J., Sanders P., Nattel S., Lau D.H. (2021). Biomarkers in Atrial Fibrillation: Pathogenesis and Clinical Implications. Card. Electrophysiol. Clin..

[39] Simonato M., Baritussio A., Carnielli V.P., Vedovelli L., Falasco G., Salvagno M., Padalino M., Cogo P. (2018). Influence of the type of congenital heart defects on epithelial lining fluid composition in infants undergoing cardiac surgery with cardiopulmonary bypass. Pediatr. Res..

[40] Wu M.Y., Yiang G.T., Liao W.T., Tsai A.P., Cheng Y., Cheng P., Li C., Li C. (2018). Current Mechanistic Concepts in Ischemia and Reperfusion Injury. Cell Physiol. Biochem..

[41] Farías J.G., Herrera E.A., Carrasco-Pozo C., Sotomayor-Zárate R., Cruz G., Morales P., Castillo R.L. (2016). Pharmacological models and approaches for pathophysiological conditions associated with hypoxia and oxidative stress. Pharmacol. Ther..

[42] Bai J., Lyden P.D. (2015). Revisiting cerebral postischemic reperfusion injury: New insights in understanding reperfusion failure, hemorrhage, and edema. Int. J. Stroke.

[43] Himmetoglu S., Dincer Y., Bozcali E., Vural V.A., Akcay T. (2009). Oxidative DNA damage and antioxidant defense after reperfusion in acute myocardial infarction. J. Investig. Med..

[44] Schipper D.A., Marsh K.M., Ferng A.S., Duncker D.J., Laman J.D., Khalpey Z. (2016). The Critical Role of Bioenergetics in Donor Cardiac Allograft Preservation. J. Cardiovasc. Transl. Res..

[45] Nakamura K., Miura D., Kusano K.F., Fujimoto Y., Sumita-Yoshikawa W., Fuke S., Nishii N., Nagase S., Hata Y., Morita H. (2009). 4-Hydroxy-2-nonenal induces calcium overload via the generation of reactive oxygen species in isolated rat cardiac myocytes. J. Card. Fail..

[46] Yu Y., Yan Y., Niu F., Wang Y., Chen X., Su G., Liu Y., Zhao X., Qian L., Liu P. (2021). Ferroptosis: A cell death connecting oxidative stress, inflammation and cardiovascular diseases. Cell Death Discov..

[47] Duez H., Pourcet B. (2021). Nuclear Receptors in the Control of the NLRP3 Inflammasome Pathway. Front. Endocrinol..

[48] Gunata M., Parlakpinar H. (2021). A review of myocardial ischaemia/reperfusion injury: Pathophysiology, experimental models, biomarkers, genetics and pharmacological treatment. Cell Biochem. Funct..

[49] Mora-Ruíz M.D., Blanco-Favela F., Rueda A.K., Legorreta-Haquet M.V., Chávez-Sánchez L. (2019). Role of interleukin-17 in acute myocardial infarction. Mol. Immunol..

[50] Baci D., Bosi A., Parisi L., Buono G., Mortara L., Ambrosio G., Bruno A. (2020). Innate Immunity Effector Cells as Inflammatory Drivers of Cardiac Fibrosis. Int. J. Mol. Sci..

[51] Nattel S., Heijman J., Zhou L., Dobrev D. (2020). Molecular Basis of Atrial Fibrillation Pathophysiology and Therapy: A Translational Perspective. Circ. Res..

[52] Kobara M., Naseratun N., Toba H., Nakata T. (2021). Preconditioning with Short-term Dietary Restriction Attenuates Cardiac Oxidative Stress and Hypertrophy Induced by Chronic Pressure Overload. Nutrients.

[53] Radovits T., Korkmaz S., Mátyás C., Oláh A., Németh T.B., Páli S., Hirschberg K., Zubarevich A., Gwanmesia P.N., Li S. (2015). An altered pattern of myocardial histopathological and molecular changes underlies the different characteristics of type-1 and type-2 diabetic cardiac dysfunction. J. Diabetes Res..

[54] Sultan A., Singh J., Howarth F.C. (2020). Mechanisms underlying electro-mechanical dysfunction in the Zucker diabetic fatty rat heart: A model of obesity and type 2 diabetes. Heart Fail. Rev..

[55] Castillo R.L., Arias C.A., Farías J.G. (2014). Omega 3 chronic supplementation attenuates myocardial ischaemia-reperfusion injury through reinforcement of antioxidant defense system in rats. Cell Biochem. Funct..

[56] Oliveira M.S., Tanaka L.Y., Antonio E.L., Brandizzi L.I., Serra A.J., Dos Santos L., Krieger J.E., Laurindo F.R., Tucci P. (2020). Hyperbaric oxygenation improves redox control and reduces mortality in the acute phase of myocardial infarction in a rat model. Mol. Med. Rep..

[57] Xu Y., Guo W., Zeng D., Fang Y., Wang R., Guo D., Qi B., Xue Y., Xue F., Jin Z. (2021). Inhibiting miR-205 Alleviates Cardiac Ischemia/Reperfusion Injury by Regulating Oxidative Stress, Mitochondrial Function, and Apoptosis. Oxid. Med. Cell Longev..

[58] Ndrepepa G. (2019). Myeloperoxidase—A bridge linking inflammation and oxidative stress with cardiovascular disease. Clin. Chim. Acta.

[59] Farías J.G., Molina V.M., Carrasco R., Zepeda A.B., Figueroa E., Letelier P., Castillo R.L. (2017). Antioxidant Therapeutic Strategies for Cardiovascular Conditions Associated with Oxidative Stress. Nutrients.

[60] Chen M., Li X., Mu G. (2022). Myocardial protective and anti-inflammatory effects of dexmedetomidine in patients undergoing cardiovascular surgery with cardiopulmonary bypass: A systematic review and meta-analysis. J. Anesth..

[61] Antonic M., Lipovec R., Gregorcic F., Juric P., Kosir G. (2017). Perioperative ascorbic acid supplementation does not reduce the incidence of postoperative atrial fibrillation in on-pump coronary artery bypass graft patients. J. Cardiol..

[62] Del Campo A., Perez G., Castro P.F., Parra V., Verdejo H.E. (2021). Mitochondrial function, dynamics and quality control in the pathophysiology of HFpEF. Biochim. Biophys. Acta Mol. Basis Dis..

[63] Bayes-Genis A., Bisbal F., Núñez J., Santas E., Lupón J., Rossignol P., Paulus W. (2020). Transitioning from Preclinical to Clinical Heart Failure with Preserved Ejection Fraction: A Mechanistic Approach. J. Clin. Med..

[64] Petersen F., Rodrigo R., Richter M., Kostin S. (2017). The effects of polyunsaturated fatty acids and antioxidant vitamins on atrial oxidative stress, nitrotyrosine residues, and connexins following extracorporeal circulation in patients undergoing cardiac surgery. Mol. Cell Biochem..

[65] Matata B.M., Elahi M.M. (2019). In Situ Oxidative Stress and Atrial Cell Deaths in Patients with Valve Disease. Cardiovasc. Hematol. Disord. Drug Targets.

[66] Abuelgasim E., Shah S., Abuelgasim B. (2021). Clinical overview of diabetes mellitus as a risk factor for cardiovascular death. Rev. Cardiovasc. Med..

[67] Kim H.M., Cho G., Hwang C., Choi H., Park J.-B., Yoon Y.E., Kim H. (2018). Myocardial Strain in Prediction of Outcomes After Surgery for Severe Mitral Regurgitation. JACC Cardiovasc. Imaging.

[68] Ahmed M.I., Gladden J.D., Litovsky S.H., Lloyd S.G., Gupta H., Inusah S., Denney Jr T., Powell P., McGiffin D.C., Dell’Italia L.J. (2010). Increased oxidative stress and cardiomyocyte myofibrillar degeneration in patients with chronic isolated mitral regurgitation and ejection fraction >60%. J. Am. Coll. Cardiol..

[69] Dhalla N.S., Elimban V., Bartekova M., Adameova A. (2022). Involvement of Oxidative Stress in the Development of Subcellular Defects and Heart Disease. Biomedicines.

[70] Abbate A., Toldo S., Marchetti C., Kron J., Van Tassell B.W., Dinarello C. (2020). Interleukin-1 and the Inflammasome as Therapeutic Targets in Cardiovascular Disease. Circ. Res..

[71] Potere N., Bonaventura A., Abbate A. (2024). Novel Therapeutics and Upcoming Clinical Trials Targeting Inflammation in Cardiovascular Diseases. Arterioscler. Thromb. Vasc. Biol..

[72] Ridker P.M., Everett B.M., Thuren T., MacFadyen J.G., Chang W.H., Ballantyne C., Fonseca F., Nicolau J., Koenig W., Anker S.D. (2017). Antiinflammatory therapy with canakinumab for atherosclerotic disease. N. Engl. J. Med..

[73] Van Tassell B.W., Michael J., Appleton L.D., Roberts C.S., Kontos M.C., Abouzaki N., Melchior R., Mueller G., Garnett J., Canada J. (2018). Rationale and design of the Virginia Commonwealth University-Anakinra Remodeling Trial-3 (VCU-ART3): A randomized, placebo-controlled, double-blinded, multicenter study. Clin. Cardiol..

[74] Yao C., Veleva T., Scott L., Cao S., Li L., Chen G., Jeyabal P., Pan X., Alsina K.M., Abu-Taha I. (2018). Enhanced Cardiomyocyte NLRP3 Inflammasome Signaling Promotes Atrial Fibrillation. Circulation.

[75] Xing Y., Yan L., Li X., Xu Z., Wu X., Gao H., Chen Y., Ma X., Liu J., Zhang J. (2023). The relationship between atrial fibrillation and NLRP3 inflammasome: A gut microbiota perspective. Front. Immunol..

[76] Zhang B., Hou J., Liu J., He J., Gao Y., Li G., Ma T., Lv X., Dong L., Yang W. (2025). Hydrogen decreases susceptibility to AngII-induced atrial fibrillation and atrial fibrosis via the NOX4/ROS/NLRP3 and TGF-β1/Smad2/3 signaling pathways. PLoS ONE.

[77] Narendran S., Pereira F., Ambati J. (2020). NLRP3 Inflammasome Inhibition: A Potential Therapeutic Strategy to Attenuate Postinfarction Adverse Cardiac Remodeling. JACC Basic Transl. Sci..

[78] Wei Z., Fei Y., Wang Q., Hou J., Cai X., Yang Y., Chen T., Xu Q., Wang Y., Li Y. (2021). Loss of Camk2n1 aggravates cardiac remodeling and malignant ventricular arrhythmia after myocardial infarction in mice via NLRP3 inflammasome activation. Free Radic. Biol. Med..

[79] Cheng X., Zhao H., Wen X., Li G., Guo S., Zhang D. (2023). NLRP3-inflammasome inhibition by MCC950 attenuates cardiac and pulmonary artery remodelling in heart failure with preserved ejection fraction. Life Sci..

[80] Sadeghi M., Khosrawi S., Heshmat-Ghahdarijani K., Gheisari Y., Roohafza H., Mansoorian M., Hoseini S. (2020). Effect of melatonin on heart failure: Design for a double-blinded randomized clinical trial. ESC Heart Fail..

[81] Hoseini S.G., Heshmat-Ghahdarijani K., Khosrawi S., Garakyaraghi M., Shafie D., Roohafza H., Mansourian M., Azizi E., Gheisari Y., Sadeghi M. (2021). Effect of melatonin supplementation on endothelial function in heart failure with reduced ejection fraction: A randomized, double-blinded clinical trial. Clin. Cardiol..

[82] Vashi R., Patel B.M. (2021). NRF2 in Cardiovascular Diseases: A Ray of Hope. J. Cardiovasc. Transl. Res..

[83] Quiles J.M., Pepin M.E., Sunny S., Shela S.B., Challa A.K., Dalley B., Hoidal J.R., Pogwizd S.M., Wende A.R., Rajasekaran N.S. (2021). Identification of Nrf2-responsive microRNA networks as putative mediators of myocardial reductive stress. Sci. Rep..

[84] Sairam T., Patel A.N., Subrahmanian M., Gopalan R., Pogwizd S.M., Ramalingam S., Sankaran R., Rajasekaran N.S. (2018). Evidence for a hyper-reductive redox in a sub-set of heart failure patients. J. Transl. Med..

[85] Carnes C.A., Janssen P.M., Ruehr M.L., Nakayama H., Nakayama T., Haase H., Bauer J.A., Chung M.K., Fearon I.M., Gillinov A.M. (2007). Atrial glutathione content, calcium current, and contractility. J. Biol. Chem..

[86] Rennison J.H., Li L., Lin C.R., Lovano B.S., Castel L., Wass S.Y., Cantlay C.C., McHale M., Gillinov A.M., Mehra R. (2021). Atrial fibrillation rhythm is associated with marked changes in metabolic and myofibrillar protein expression in left atrial appendage. Pflugers Arch..

[87] Carnes C.A., Chung M.K., Nakayama T., Nakayama H., Baliga R.S., Piao S., Kanderian A., Pavia S., Hamlin R.L., McCarthy P.M. (2001). Ascorbate attenuates atrial pacing-induced peroxynitrite formation and electrical remodeling and decreases the incidence of postoperative atrial fibrillation. Circ. Res..

[88] Antoniades C., Demosthenous M., Reilly S., Margaritis M., Zhang M.-H., Antonopoulos A., Marinou K., Nahar K., Jayaram R., Tousoulis D. (2012). Myocardial redox state predicts in-hospital clinical outcome after cardiac surgery effects of short-term pre-operative statin treatment. J. Am. Coll. Cardiol..

[89] Winter J.L., Castro P.F., Quintana J.C., Altamirano R., Enriquez A., Verdejo H.E., Jalil J.E., Mellado R., Concepción R., Sepúlveda P. (2014). Effects of trimetazidine in nonischemic heart failure: A randomized study. J. Card. Fail..

[90] Ocaranza M.P., Moya J., Jalil J.E., Lavandero S., Kalergis A.M., Molina C., Gabrielli L., Godoy I., Córdova S., Castro P. (2020). Rho-kinase pathway activation and apoptosis in circulating leucocytes in patients with heart failure with reduced ejection fraction. J. Cell Mol. Med..

[91] Ternacle J., Berry M., Alonso E., Kloeckner M., Couetil J.-P., Randé J.-L., Gueret P., Monin J.-L., Lim P. (2013). Incremental value of global longitudinal strain for predicting early outcome after cardiac surgery. Eur. Heart J. Cardiovasc. Imaging.

[92] Ikeda M., Niinami H., Morita K., Saito S., Yoshitake A. (2024). Long-term results following off-pump coronary-artery bypass grafting in left ventricular dysfunction. Heart Vessels.

[93] Erturk O., Keles N., Karaagac A., Arslanhan A.S., Pocan Y.K., Yesilkaya M.I., Bozkurt B., Aydogan H., Kaplan M. (2024). Comparison of early postoperative left ventricular function with 3d ef and strain measurements according to graft selection. J. Cardiothorac. Surg..

[94] Silva R.R.M., Hueb W., Lima E.G., Rezende P.C., Soares P.R., Ramires J.A., Filho R.K. (2022). Long-term analysis of ventricular function in patients with symptomatic coronary disease who underwent on-pump or off-pump coronary artery bypass grafting. J. Cardiothorac. Surg..

[95] Downey M.C., Hooks M., Gravely A., Naksuk N., Buelt-Gebhardt M., Carlson S., Tholakanahalli V., Adabag S. (2022). Perioperative changes in left ventricular systolic function following surgical revascularization. PLoS ONE.

[96] Olsen F.J., Lindberg S., Pedersen S., Iversen A., Davidovski F.S., Galatius S., Fritz-Hansen T., Gislason G.H., Søgaard P., Møgelvang R. (2021). Global longitudinal strain predicts cardiovascular events after coronary artery bypass grafting. Heart.

[97] Wakefield B.J., Artis A.S., Alfirevic A., Sale S., Duncan A.E. (2022). Post-cardiopulmonary bypass longitudinal strain provides higher prognostic ability than baseline strain or change in strain. Ann. Card. Anaesth..

