# Supplementary material for: Oxidative Stress and NLRP3 Inflammasome as Markers of Cardiac Injury Following Cardiopulmonary Bypass: Potential Implications for Patients with Preoperative Heart Failure with Reduced Ejection Fraction
Source: Antioxidants (Basel). 2025 Oct 30;14(11):1311. doi: 10.3390/antiox14111311 (PMC12649429; doi:10.3390/antiox14111311)
Supplement: Supplementary file 1 [file antioxidants-14-01311-s001.zip › antioxidants-3712424-supplementary.pdf]

Full screening W. Blots.
S1.

$\alpha\beta$ tubulin

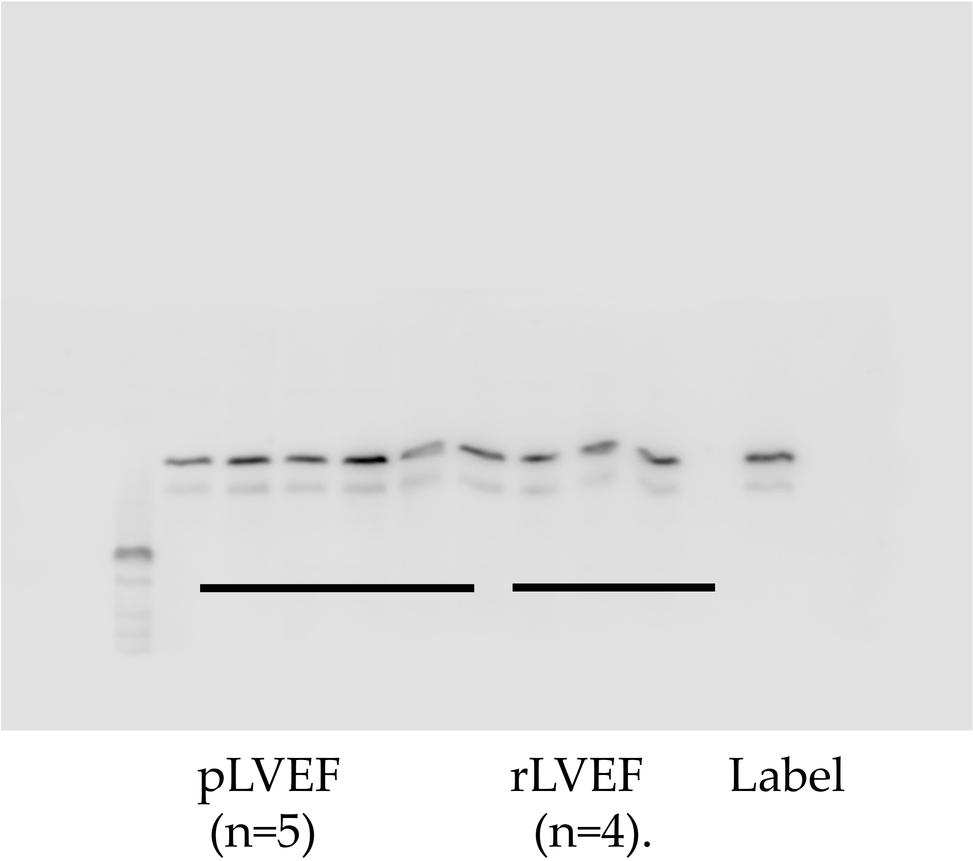

NRF2

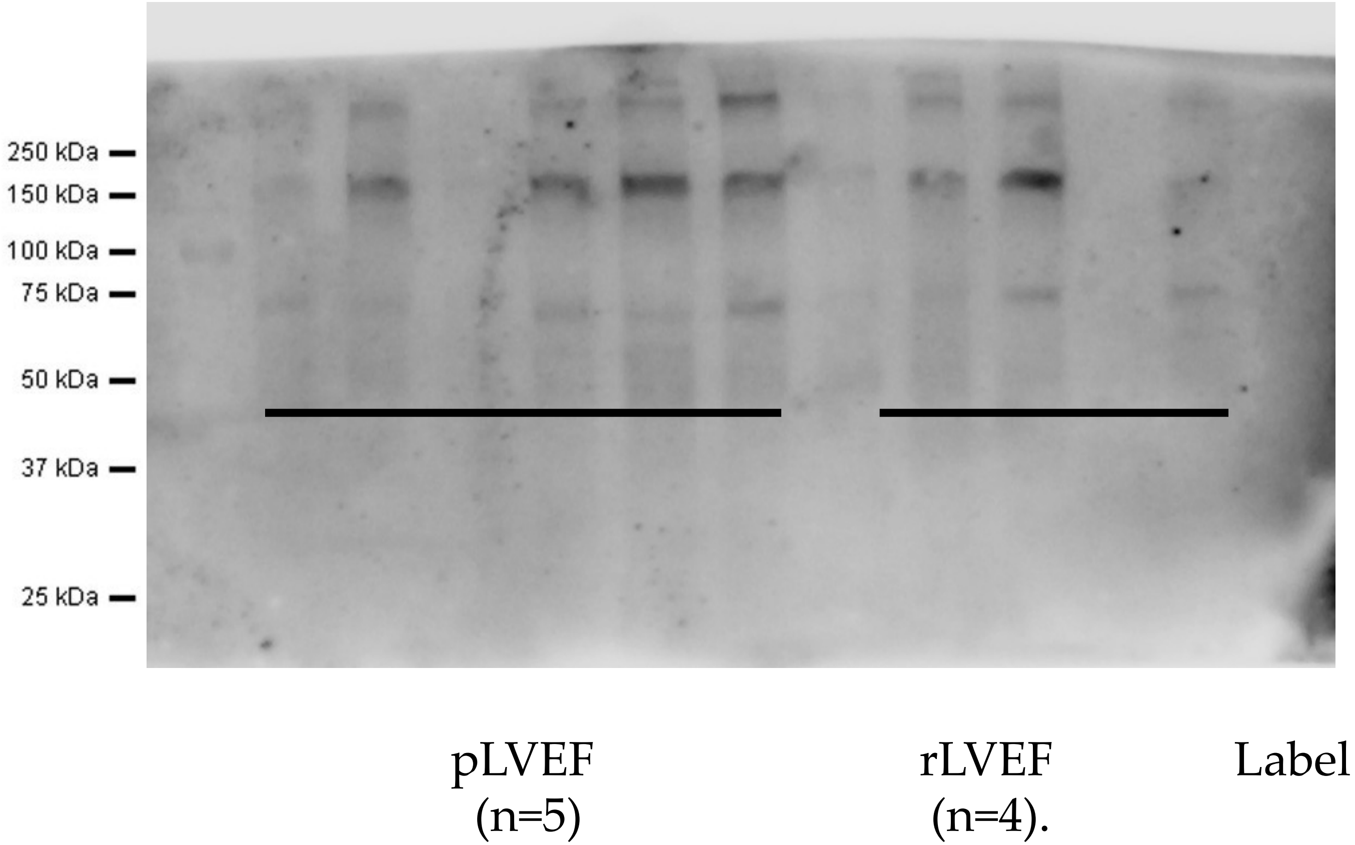

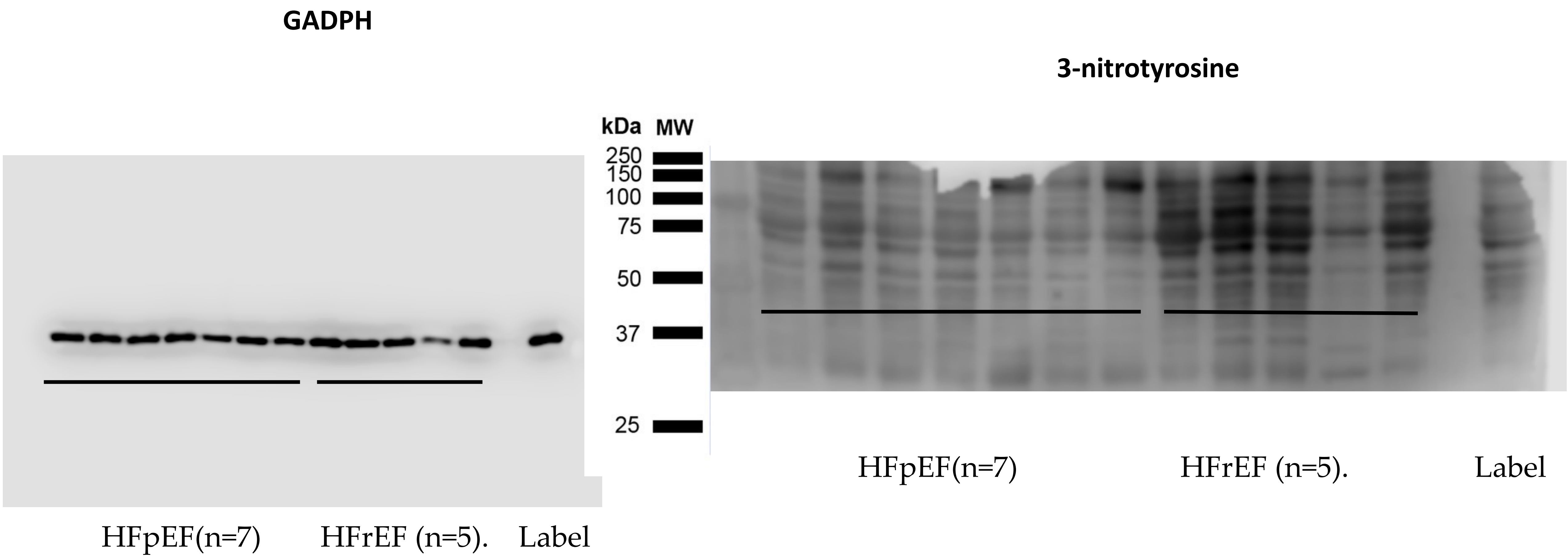
GADPH
3-nitrotyrosine
kDa MW
250
150
100
75
50
37
25
HFpEF(n=7)
HFrEF (n=5).
Label
HFpEF(n=7)
HFrEF (n=5).
Label
